# Implementing Health Policy: Lessons from the Scottish Well Men's Policy Initiative

**DOI:** 10.3934/publichealth.2015.4.887

**Published:** 2015-12-21

**Authors:** Flora Douglas, Edwin van Teijlingen, Cairns Smith, Mandy Moffat

**Affiliations:** 1 Public Health Nutrition, University of Aberdeen, Aberdeen, Scotland,; 2 Health and Social Care, University of Bournemouth, Bournemouth, England,; 3 Public Health, University of Aberdeen, Aberdeen, Scotland,; 4 Academic Primary Care, University of Aberdeen, Aberdeen, Scotland.

**Keywords:** Policy implementation, health inequalities, men's health, planning frameworks, Scotland

## Abstract

**Background:**

Little is known about how health professionals translate national government health policy directives into action. This paper examines that process using the so-called Well Men's Services (WMS) policy initiative as a ‘real world’ case study. The WMS were launched by the Scottish Government to address men's health inequalities. Our analysis aimed to develop a deeper understanding of policy implementation as it naturally occurred, used an analytical framework that was developed to reflect the ‘rational planning’ principles health professionals are commonly encouraged to use for implementation purposes.

**Methods and materials:**

A mixed-methods qualitative enquiry using a data archive generated during the WMS policy evaluation was used to critically analyze (post hoc) the perspectives of national policy makers, and local health and social care professionals about the: (a) ‘policy problem’, (b) interventions intended to address the problem, and (c) anticipated policy outcomes.

**Results and conclusions:**

This analysis revealed four key themes: (1) *ambiguity* regarding the policy problem and means of intervention; (2) *behavioral framing* of the policy problem and intervention; (3) *uncertainty* about the policy evidence base and outcomes, and; (4) a focus on *intervention as outcome*. This study found that mechanistic planning heuristics (as a means of supporting implementation) fails to grapple with the indeterminate nature of population health problems. A new approach to planning and implementing public health interventions is required that recognises the complex and political nature of health problems; the inevitability of imperfect and contested evidence regarding intervention, and, future associated uncertainties.

## 1. Introduction

Policy-driven, public health interventions are standard practice in countries with high levels of government control over health promotion budgets [1]. However, little is known about how health professionals respond to and translate national government policy directives at the local level [2],[3]. This paper aims to address that knowledge gap by presenting the findings of a study of the interpretation and implementation of a national health policy initiative, as it occurred in a real world setting, by health, social care and third sector staff responding to a government policy directive aimed at improving men's health in Scotland. This naturally occurring phenomenon was captured, considered and compared against the principles of so-called ‘rational planning’ approaches which health promotion/public health professionals are commonly encouraged to use to design, implement and evaluate health improvement interventions [4]–[7]. The analysis revealed some specific challenges and tensions for professionals (and researchers) planning interventions aimed at addressing or ameliorating complex, indeterminate health and social problems, such as men's health inequalities, using orthodox, linear planning approaches.

While considerable energy has been directed toward the ‘research into policy’ stage [8],[9], there has been much less attention given to policy interpretation and implementation, and the ‘delivery’ groups upon whom researchers and policy makers depend for successful implementation and impact [10]. The translation of policy into frontline action is erroneously regarded as a relatively objective and unproblematic part of the ‘policy process’ [10],[11]. Indeed, professionals as intended policy implementers are often incorrectly characterised as passive recipients of policy directives, who are thought to make policy happen ‘in the field’ just as the policy makers intended at the design stage [12]. Some have argued that frontline staff (re)interpret policy objectives according to their personal or local organisational objectives and problem priorities [13]–[15]. It is crucial then to understand the dynamics that surround policy interpretation to enhance the usefulness of policy interventions applied to improve population health [16],[17]. Becoming more familiar with the ‘normative worlds’ of policy makers and practitioners is key to that understanding [10].

This paper examines the process of real world policy interpretation and implementation surrounding a government policy directive, the Well Men's Services (WMS) policy initiative launched in 2004 aimed at addressing Scottish men's health inequalities [18]. It does so using an analysis of archived research data that was generated during an external evaluation of the WMS policy and its implementation, which took place during 2005–2007 [19]. Before presenting the results of this analysis, it is important to understand the context within which the WMS policy emerged.

Health inequalities and concern to address health inequalities has been a policy issue for successive Scottish governments over several decades [20]. Scottish men live on average 2.4 years less than those in other parts of the United Kingdom (UK), 75.0 years compared to a UK average of 77.5 years in 2008 [21]. Male mortality rates in Scotland are poor even compared to areas of Eastern Europe [22]. Within Glasgow, there is a 15 year difference in life expectancy between the most deprived and the least deprived areas (63.5 and 78.7 years respectively) [23]. Notwithstanding variations in mortality and morbidity that can be explained by biological differences between men and women, there are a range of overlapping and contested explanations about the underlying causes of men's health inequalities [24],[25].

The most popular *(behavioral/cultural)* explanation of men's health inequalities is associated with men's deficient lifestyle health behaviors. Seen from this perspective men make sub-optimal use of health services compared to women. Thus men are less likely to report illness [26]; tend to make less use of primary health care services; delay seeking advice about health problems unless urgent [27],[28] and are believed to suffer poorer health outcomes as a result of this behavior [29],[30]. At the same time, there was little published evidence on how to improve men's uptake of services [31]. And while there are differences noted in the Scottish context, between men and women in relation to health outcomes associated with for example cardiovascular disease, cancer, diabetes and multiple sclerosis, it is argued that it is the way women and men relate to their health through differences in power, opportunities, and personal and social expectations that play into these outcomes. For example men are more likely to rate their health good or very good compared to women, yet are more likely to pursue risky occupations compared to women, and risky health behaviors [32].

The *material/structural* explanation argues that it is unjust social structures which constrain access to material and economic resources that creates the burden of ill health, and are largely borne by the poor [33]. While there is little debate that material deprivation is associated with ill-health, this is a rarer explanation for men's health inequalities [24]. MacDonald (2006) has similarly highlighted the lack of interest in material/structural causes related to men's health inequalities [34]. This lack of ‘socio-economic’ focus is thought to arise from being perceived men (in general) as the more economically and socially advantaged group in society [35], a stance increasingly challenged [36],[37]. Related to this perspective is the *psychosocial stress* explanation which views health inequalities as something that occurs not only due to constrained access to economic resources, but is magnified by the experience of relative deprivation associated with one's position in the social hierarchy. From this perspective, health problems are created through psychosocial processes that work at individual and societal levels [24]. The psychosocial effect of unequal incomes is thought to negatively impact individual self-esteem, social cohesion and trust, resulting in more individuals turning to unhealthy ‘coping’ behaviors such as smoking or excessive alcohol consumption [38],[39]. Accordingly, Hunt and colleagues have argued that the impact of being economically and social unequal to other men (and to some women) has the potential to generate poor self-esteem which directly effects men's health, and cause some to engage in “extreme macho” health damaging behaviors to regain their social status through “*appealing to hierarchies of masculinity rather than hierarchies of social class”* [40].

It was against this backdrop of contested causal accounts of men's health inequalities - ranging from individually focused to structural explanations - that the WMS policy initiative was funded by the Scottish Government to address men's health inequalities. The main logic and stated goals of the WMS initiative were that men's health would be improved by encouraging them to adopt healthier lifestyles, with a particular focus on their making more effective use of health and social care services. The health status of so-called ‘hard-to-reach’ and economically disadvantaged men was of particular concern, and the funded initiatives were specifically expected to engage with these groups. This policy initiative was launched within a wider health promotion and social inclusion policy context concerned with reducing inequalities [41],[42],[43]. But the specific rational and evidence cited as underpinning the WMS policy, that was evident in the related policy documentation, was centered on a nurse-led men's health promotion programme that had emerged within Scotland within the previous 2 years [44],[45].

As part of a commissioning process, a series of workshops were held by health department policy makers to raise awareness about the WMS aims and intended outcomes throughout all Scottish health board areas, before they invited submissions for 2.5 years of ring fenced funding from interested parties. Ultimately, seven multi-agency coalitions (involving health, social care and voluntary sector organisations) were offered funding to develop and implement the local programme proposals that were perceived to fit the policy aims. Ultimately, seven multi-agency coalitions (involving health, social care and voluntary agency professionals), located in a mixture of urban, rural and remote island areas were selected for this government funding. The successful coalitions had offered to develop and deliver sixteen separate projects. These varied in terms of intended target groups, settings, health topics, and intervention strategies. Some of the funded projects were developments of existing community based work, while others were completely new initiatives.

## 2. Methods and materials

Using an earlier evaluation of the WMS policy initiative [44], we applied a mixed-methods qualitative post hoc analysis of national policy maker, and local health and social care professionals' perspectives about the: (a) ‘policy problem’; (b) interventions/proposed solutions intended to address the problem; and the (c) anticipated intervention outcomes. The overall aim of this analysis was to investigate how the process of policy implementation *happened* in a real world setting compared to the principles of what is *supposed to happen* according to rational planning tenets [4]–[7].

Such rational planning approaches have emerged not only in the population health science but also in the grey non-governmental organization literature over recent decades [46],[47], much of it driven by a concern to optimize programme or policy implementation and effectiveness [48]. *S*uch mechanistic planning models assume that it is possible to identify, describe and analyze all possible components of a social system, the means of changing aspects of that system that would improve it, and then choose the most effective course of action from a range of possible alternatives [49]. It also assumes a linear relationship between inputs and outputs, and that combined action of different inputs can be figured out and predicted [50],[51]. Despite some criticism of the ability of (what has been described as reductionist and mechanistic) thinking to address complex population health issues [49]–[51], this approach remains popular with professional organizations and public health bodies to this day in the UK [52].

Re-analyzing historical data can offer more insight and knowledge of social patterns and processes informing contemporary debates on similar issues [53]. The WMS data archive comprised (a) eleven key documents that used to communicate the policy makers ideas and desired outcomes regarding the local delivery of the policy, as well as the detailed local health board WMS plans that were generated in response to the policy directive and (b) interview data and field note data that had recorded the perspectives and experiences of 42 health, social care and third sector managers and professionals from across Scotland who had played a major role in designing and implementing local interventions.

### 2.1. Secondary analysis of interview data

The qualitative approach used to investigate implementer perspectives during the original evaluation had been conducted using Grounded Theory principles [54]. Face-to-face, semi-structured interviews had been conducted using a specially designed topic guide to explore interviewees' experiences of the conceptual and practical development of, and aspirations for their locally developed WMS interventions. All interviews were audio-recorded and anonymously transcribed verbatim. Twenty eight interview transcripts originated from NHS (National Health Service) employees and 14 from employees of a range of partner voluntary and statutory organizations.

Framework analysis was used to re analyze these data. This involved a rigorous process of data familiarization, identification of concepts and themes, data summarizing, synthesis, description and interpretation [55]. The analysis was conducted by the first author, in conjunction with one of co-authors who independently reviewed and discussed all themes as the analysis proceeded. Emerging interpretations and conclusions were constantly checked against the original data and field notes.

Interpretative documentary analysis has widely been used in health-policy analysis [56],[57]. This process enables researcher's access to traces of the past which, through the process interpretive analysis, helps to reconstruct that past to inform the present [58]. The documentary analysis was conducted in discreet stages [57]. The documents (which varied in length from two to 12 pages) were sorted into two sets; policy maker (PM) generated and policy implementer (PI) generated. This process enabled the graphical display and assessment of common and exceptional assumptions that related to the perceived problem, its determinants, and proposed interventions, and anticipated outcomes.

### 2.2. Integrative analysis

Once both the documentary and secondary interview analyses had been completed separately, cross-cutting common and contradictory themes that emerged within and between the interview and documentary records were highlighted. Anonymised, verbatim quotes illustrate the findings.

Ethical approval was awarded for the WMS evaluation by the Scotland A Research Ethics Committee. All participants gave informed consent and agreed for their interviews to be audio-taped.

## 3. Findings

This section presents the themes that emerged as they pertained to PMs and PIs conceptions of the policy problem (its nature, determinants and groups of particular concern), the proposed interventions, and their anticipated outcomes.

### 3.1. The men's health issue

Ambiguity was ubiquitous in both the policy directive and implementers' responses. Both the PM and PI documentation lacked detail about what health issues were to be improved, or which aspects of inequalities were to be reduced. The PM documentation contained statements about the WMS policy being launched due to the “need to improve men's health in Scotland” and to “reduce health inequalities”. The PI documentation focused more on men's deficient behaviours (poor lifestyle behaviours/lack of health service use) or their status, i.e. as socially excluded or ‘hard-to-reach’. In some areas the term hard-to-reach' was used to denote specific subgroups of men, and in other areas it was used to describe all men.

Analysis of the interview data revealed that a health (or ill-health) problem was only one of five issues that had been used as the basis for the local implementation plans. Other factors included, health professionals' past experiences about working with men, beliefs about interventions that men required to improve their health, local advocacy and lobbying by community groups regarding male only health services, and the opportunity to gain funding for services that were already in place or had been planned for some time. It was notable in fact that some plans described solutions as the basis for their plans rather than problems per se. Where discussed, the (ill) health problem was also variously and rather vaguely described. PI informants (across the different professional groups) commonly cited examples using national epidemiological-type data to make comparisons about Scottish male mortality at a national level with countries in across Europe, thought to be doing better than Scotland. Other views of the problem included beliefs about: unhealthy lifestyle and health care seeking behaviour patterns amongst men; local area deprivation; the existence of undiagnosed disease (such as high blood pressure or heart disease. Views about inadequate health service provision for men were also commonplace amongst health care informants. Several issues are illustrated by this example:

*Well we had been aware that men weren't using the general mainstream health services with our area. But also when you look at the statistics of men's health within … it was really we thought we need to look at some of innovative ways of actually getting men through the door or accessing men to help them be aware of health issues so that they could then address them themselves* .. P30 (Voluntary sector manager)

The PM perspective described men in terms of being “hard-to-reach”; and “socially-excluded”; but also those who were described as “lack[ing] concern about their health or lifestyle”. It was notable that numbers or estimates of men described as such were lacking in these descriptions.

Local intervention plans indicated that a wide variety of groups were the subject of concern according to PI perspectives. Target groups were variously defined in terms of age; sexuality; employment status; ethnic background; locality (which was commonly described in terms of economic deprivation status or remoteness); or last visit to GP (General Practitioner). Most plans provided long lists of groups of men who were to be targeted with local interventions, and little information about the numbers or proportions of men to be targeted.

The interview data revealed a similar lack of clarity about the intended policy beneficiaries and in some areas, varying degrees of uncertainty, illustrated by this health service manager, who when outlining the local target group described:

*....., young men, unemployed men, homeless men because we have got quite a significant proportion of homeless men in our area and men from black community, and we are also going to target some GP practices. We are just targeting everyone at the moment to ascertain if by sending men in their practice who have not attended their GP for maybe 2 years, maybe 5 years depending on what the search shows.... So at the moment I can't be so specific about the actual target group..* (P35 NHS employed public health nurse)

Furthermore, ideas about which groups would be targeted shifted quite regularly within local areas. Most informants described instances of local meetings where various ideas had been discussed. In a minority of cases disagreement had emerged about which groups to target and in other areas different ideas were held within the same localities about who to target.

The documented policy perspectives located the causes of the men's health problem in men's (deficient) health behaviours. There was a perceived need to; “….encourage men to consider their own health and to understand the impact on them of the lifestyle choices they have made” and to “…capture their interest and help them to understand their lifestyle choice”. There were references to wider determinants of health, such as poverty and unemployment, but these issues were generally expressed in terms of their being the background context, with men's poor health being more directly linked to behavioral or attitudinal deficits.

In contrast, the local PI plans contained many references to wider determinants of men's health i.e. deprivation and homelessness, and curiously, no statements about behavioural issues. Yet, the individual interview accounts were dominated by behavioural explanations. Most commonly, men's poor health were linked with their faulty attitudes and behaviour as above. Men were commonly portrayed to be unwittingly pursuing poor lifestyle behaviours, or reluctance to seek help from others to improve it, or reduce their risk of further or future ill-health. These perceived negative attitudes were explained by some as a fear of becoming emasculated by seeking help, or, because of Scottish cultural attitudes regarding men and their underuse of health services:

*Yeah I think medical stuff might scare a lot of guys. We did a bit of research … we interviewed homeless on the street and what came out of that was very clearly that they saw them as going to the doctors as an attack on their masculinity, any contact with medical services was* (P12 NHS employed public health lead)

There was a prevalent view that such attitudes were modifiable through professional intervention.

### 3.2. Notions of solutions

The PM documentation contained explicit and multiple intervention/solution expectations. There was particular interest and importance placed on engaging men in the process of designing and delivering local interventions. The PM documentation expressed a clear desire to see a so-called ‘community development’ type approach implemented and required local WMS programme to engage with men in their communities and empower them to develop innovative, community-led solutions. Men were portrayed as key partners from this perspective, with an emphasis on the need to “identify what the community needs and wants”; “understand their needs and priorities and how we can better serve them”; and to “engage with different groups of men in their own communities”. Furthermore, developing partnership arrangements with local communities and existing community structures (including third sector and social care organisations) were also described as key PM requirements.

At the same time, PIs were given very specific direction and encouragement to implement a professional, nurse-led health screening intervention, which had been the central aspect of an existing model of practice that was operating in one region of Scotland at the time. Implementers were instructed to: “consider where and how they undertake health assessments”; and, the precise tools that were to be used by local interventions, including the health assessment protocol to be used.

Despite the apparent policy steer encouraging implementers to develop innovative community-determined interventions, implementers responded most obviously to the directive about providing nurse-led screening interventions. All area health board plans described an almost exclusive focus on providing individual health check assessments, citing the suggested model as the central aspect of their intervention proposals. Five documents described intentions to link the clinics with “supporting” agencies; referred to as ‘signposting’ by some projects. Examples of supporting agencies given included welfare, housing, and healthy living centres. Statements about conducting community development work to engage men were present in only two plans.

The notion of providing health checks dominated the interview data too, both in terms of the conceptions of solutions that some informants had described when discussing the key factors that had influenced the early development of local plans, and was present in the majority of implementers' notions of evidence that had informed the development of their interventions. This example is typical:

*… obviously the Well Man Clinics is a very specific way of men going in to speak about their health. Our role in that is more about really trying to support men in going to the Well Man's Clinics* (P5 NHS employed primary care nurse)

PIs were asked about the evidence base for their work, many either knew that evidence did not exist, because they believed that the research had not been conducted, or, they were uncertain if it existed or not, described thus:

*I am aware that the (x) project has been evaluated and that is held up as a role model because it has been running now for quite a few years. There have been other clinics or services within Scotland but I have not actually seen any paperwork as to how they have been evaluated……And what the outcomes were* (P30 Voluntary sector manager)

Many were uncertain about the robustness of the evidence base about what works to improve health for men per se, and about the (lack of) evidence that was perceived to exist about health check type interventions, which was the main approach that local programmes were planning to use or were using at the time the interviews were conducted, illustrated by this example:

*Well that's the problem isn't it? There is very little research ... it's all mainly best practice .. ..So hopefully as we develop services, audit services, research questions will start popping up and we will .. have a much firmer evidence base for what we are doing… I think it's quite interesting ‘cause your caught in this dilemma, you know it is supposed to be community development that we are doing and yet the model that we are using with the men's health clinic here is very medical* (P40 NHS employed public health nurse)

A few did talk about instigating community development work alongside health check work, but this was very largely associated with encouraging men to use health check clinics.

### 3.3. Views about policy outcomes

Within the policy documentation no obvious statements were found about expectations about the impact sought on any specific health outcomes from the local WMS interventions, although local projects were urged to “collect data during the project”. Similarly, analysis of the local implementation plans revealed no explicit statements about anticipated outcomes, although all contained some sentences about conducting local evaluations.

Towards the end of each interview PIs were asked about expectations of their local interventions over the short, medium and longer term. The most commonly discussed outcome by far, was associated with having established and still be delivering some form of mainstream primary health care service for men, illustrated thus:

*My ultimate aim is to have I guess 3 years down the road because we are thinking we will get an extra couple of years funding out of it would be to have these imbedded very securely* …. (P27 NHS employed primary care nurse)

More exceptionally, the notion that primary care adopts and embeds a more ‘gender-sensitive’ approach within existing work was considered an appropriate outcome from this policy.

The analysis also revealed that individual implementers had been interested in achieving changed attitudes to male health seeking behaviour and the need for a male only service, with lay and professional groups, as long term outcomes of their intervention, e.g.:

*…and long term being about 20 years into the future I suppose when my wee boy that has just been born is 10 year old or 12 year old and going to high school … there is a lot of work been going on with our school health service and the input fae health visitors and other community health practitioners into schools I suppose we know what em school age kids think about and all the stuff around men's health so that men not accessing services …and that can be changed* …(P19 NHS employed health visitor).

Establishment, use and developing a general acceptance of the need for a male-only primary care service appears to have been the primary goals of most interviewees. Strikingly, the issues that generated the least amount of debate about expected or (hoped for) outcomes were any specific improvements in men's health outcomes, or, establishing what men's expressed health needs were – which was a key policy aspiration.

However, the other dominant theme that emerged within discussions, particularly about longer term outcomes, were expressions of uncertainty. It was notable that early on in most interviews, PIs appeared very convinced about the need to act on the policy directive. However, they were far less certain about the outcomes that would emerge from their intervention. Uncertainty emerged in the expressed anxieties about how well local interventions would: (a) operate; (b) attract their intended beneficiaries; or, (c) have an impact on men's health outcomes. Non-health organisation informants expressed more uncertainty about these issues than health service informants. This example illustrates the disquiet one PI expressed about not only about being concerned that she might be asked about outcomes (by the interviewer), but also about what impact her local intervention might have:

*I wondered if you would ask the kind of complicated thing about outcome, and how many or what do we expect to achieve or anything like that. But obviously that is all set out in the kind of aims of it. But I wondered…If you would ask more about, you know, do I see us meeting our aims and things and things like that…. Cause I don't know* (P17 NHS employed primary care nurse).

This interviewee from voluntary organisation conveys her uncertainty about the likely uptake of her local intervention by men, and, about how the intervention might work in practice:

*… I don't know if a weekly well men's health clinic will work. I have not experienced that before. It is not something, you know… we don't think to have six months weekly men's health events so I just don't know from a lack of experience in that area. I just don't know how it will work. I don't know how many men will come in the door* (P43 Manager voluntary sector)

Some described not only their own uncertainty about outcomes, but also described being aware of the differing views held amongst other members of staff, and other key local stakeholders about the nature of the problem and who was affected by it. This issue is illustrated by the following example where the interviewee believed that integrating ‘gender sensitive’ practices into existing mainstream services was the desired outcome, but he was aware that others held different views about this too:

*I think longer term than that … perhaps it should to be mainstreamed. I don't know.. there are always arguments about whether, you know, we should be setting up separate services for men in this case or whether we should be integrating them with existing services. ...I'm not sure whether it is from the steering group group's point of view but from my point of view I think sometimes you have to set up separate services* (P31 NHS employed public health lead)

## 4. Discussion

Using this case study to look at that the process of policy translation from government department to community level, with its rational planning lens, this analysis revealed four obvious themes: i.e. (1) ambiguity regarding the policy problem and policy guidance regarding means of intervention, (2) behavioral framing of the policy problem and means of intervention; (3) uncertainty about the existing evidence for intervention, and, the possible WMS policy outcomes, and; (4); a focus on intervention as main policy outcome.

### 4.1. Ambiguity

There was evident lack of precision surrounding: (1) which male health problems were of specific WMS policy concern, and (2) which men were in need of some form of intervention emanating from the policy. The PM and PI's narratives both contained ambiguous and diverse views about the ‘problem’, ranging from very different ideas about: i. male mortality and morbidity, or lifestyle behaviour trends; ii. local area deprivation status; iii. the levels of undiagnosed disease present in the local male community, or men's ignorance of their poor lifestyle behaviour; and/or iv. the (in)adequacy of health service provision for men. There were similarly diverse ideas about which groups of men were afflicted by which problem, and these ideas varied across and within local health board areas.

This phenomenon of different problem interpretations amongst professionals was also been observed by Carlisle [17]. She found that individual health board, local authority and community informants working in the same area, on the same policy implementation steering group, had very different ideas about local priorities and problems. It is argued that public agencies often express their (often multiple) goals, in ambiguous terms [12],[59],[60]. This phenomenon is particularly stark when comparing the more precise goals of business or commercial organisations according to Feldman [61].

Lee et al. (2009) argued that one of the reasons public agencies are given ambiguous goals by governments to put into action, arises from the way political institutions and policy processes operate, i.e. where reaching a compromise between rival sets of interests is necessary to move forward on a political issue [62]. And, indeed the WMS policy emerged during at time when a Labour/Liberal Democrat Coalition government was in power in Scotland. Carlisle (2001) and Oliver (2006) have both argued that (from a policy maker's perspective) the presence of ambiguity enables different interpretations to emerge, which in turn, can be aligned to different interests, which can serve multiple goals thus providing policy makers with some leeway in dealing with any potential problems that might arise from policy implementation [33],[63].

It is important to set the imprecise accounts of health problems, and target groups found in these data, against ongoing debates about the lack of the multidimensional data thought necessary for planning local health promotion interventions [64],[65]. Godin et al found that would-be public health planners experienced significant implementation challenges when there was inadequate problem definition, arguing that a lack of deep understanding of a problem led to “indeterminate” descriptions of intended actions or interventions [66]. Moreover, it is important to note in this case, that PIs were advised in the policy documentation “that they need not provide too much detail” in their submissions.

The PM documentary analysis also revealed explicit direction about policy expectations regarding how the policy problem was to be addressed. There was an unequivocal recommendation that local areas should provide a health professional-delivered health check. However, this guidance also contained additional ambiguous advice about expectations the local programmes were expected to achieve. That is, the need for PIs to engage men, community, statutory and voluntary sector organisations to form partnerships to: (a) discover men's’ views about their health needs and notions of acceptable solutions, and (b) other types of interventions defined by the local communities. While professionally delivered interventions had obviously been endorsed by the policy makers - it was evidently not presented as the only intervention solution expected. Consequently, it was interesting to observe the apparent consensus about notions of the solution(s) in all seven areas, i.e. the need for a professionally-delivered health check type intervention, which consisted of a physical check and individual lifestyle advice giving based on the results of the physical check. This seemed somewhat paradoxical given: (a) the range of different ideas had existed about the nature of the policy problem; (b) the variety of subgroups that were proposed as target groups and: (c) the the apparent scope to be innovative in designing locally-determined solutions. Educational interventions generally find more favour (within the policy context), compared to (for example) regulatory intervention [67] even if the evidence for educational interventions is less compelling than that for regulation [20].

### 4.2. Uncertainty

The health check option emerged as the common and largely only intervention approach being put in place, but there was the obvious uncertainty amongst PIs regarding the existence of appropriate evidence. Uncertainty was also clearly evident in PI's views about the operation and outcomes of their work. There was considerable anxiety about the possible levels of uptake of local programmes amongst the target group, and about possible health outcomes that might be achieved by their work. This uncertainty emerged in a stark contradiction to the apparent confidence expressed earlier in most interviews about the need to intervene in this area, and about in the solutions informants had to offer in this case. Furthermore, uncertainty and anxiety also permeated PI's ideas about future policy outcomes.

However, those tasked with addressing health inequalities experience significant challenges in dealing with the manifold theoretical, methodological and political uncertainties, in a context where evidence is often lacking, conflicting and contested [17],[68]. As the effectiveness evidence base about what worked to address men's health inequalities was incomplete [31] and disputed [69] it is perhaps unsurprising that most interviewees did not talk about the likely impact of health outcomes, or when they did, expressed uncertainty about what impact their work might have.

### 4.3. Behavioural/cultural framing

Behavioral/cultural explanations of male health inequalities dominated PM and PI perspectives, although PIs acknowledged material/structural concerns in their formal plans, There appeared to be a somewhat uncritical acceptance of behavioural/cultural view of men's health problems in the PM documentation. However, no data suggesting how many men had deficient lifestyle behaviours (not defined) or who were known not to “care enough about their health or lifestyle' was evident in any of the documentation or PIs narratives. This analysis revealed that men were viewed as implicitly sharing the same sets of characteristics, values and needs i.e. needing to acknowledge their health threatening behaviours, and needing help (from a professional) to change their behaviour. While economic and social disadvantage were mentioned in the PI plans - these issues were presented in terms of making the case for their local area to get more money to do more educational work with men. Aronowitz (2008) argued that the way in which a health problem is perceived and framed, fundamentally determines the solutions (or interventions) deemed acceptable to those responsible for funding and implementing them [70]. Front line professionals play a crucial role in policy implementation and dictate to a large extent how well policy is implemented, and the critical role that individual professional values and beliefs plays into that process has been highlighted elsewhere [10], [12], [2], [71].

Behavioural framing is consistent with contemporary UK (and many other countries) health promotion policy, which has tended to favour educational interventions over structural or social policy changes to improve health. There is a growing body of evidence to suggest individual level behaviour change focus on its own is unlikely to make significant changes to health outcomes at the population level [3],[72]–[74]. Furthermore, the efficacy of primary care-based health checks (as a means of addressing health inequalities) had been challenged at the point that the WMS policy was launched in 2005 [75],[76]. Yet, the individual lifestyle orientation remains popular to this day [77]–[79] suggesting its deep-rooted place in policy making circles.

There is also increasing recognition of the role that media plays in health policy framing, and in continuing to reinforce the view that health problems are a matter of personal responsibility, as opposed to or as well as their being caused by wider socio-economic determinants. The media are more likely to mention personal and behavioural attributions of obesity [80], as opposed wider societal determinants of causality or solution; a notion that that has been robustly challenged by the UK Cabinet Office for Science [81]. Despite the growing body of epidemiological evidence highlighting structural inequalities and health outcomes, paradoxically there is a concomitant increase in the numbers of studies focused on behavioural explanations and interventions [82].

### 4.4. Intervention as outcome

Rational planning logic is based on the assumption that planners/implementers have clear end points in mind when designing their interventions [83]. Yet, there was a stark absence of anticipated outcomes in the WMS policy per se. PI expectations about changes to specific health outcomes was also largely absent. However, the levels of uncertainty and anxiety found in the PI interviews about their hopes or fears about what would (or would not) emerge from their interventions, indicated that the end point(s) were not at all clear for those involved in implementing the WMS. Their main outcome aspirations for the WMS policy was focussed on the establishment a male specific primary care service and cultural change associated with more men engaging in earlier health care seeking behaviour. That there was less stated interest in changes to health outcomes by the PIs might be explained by the lack of evident policy clarity about the specific aspects of men's health that were to be changed or improved.

The lack of baseline information revealed by documentary analysis provided further evidence that changes to specific health outcomes were not apparently uppermost in the minds of those drawing up the local plans. Their interest and focus was associated (one might argue), with the one relatively clear policy directive i.e. the provision a health assessment. There was much less expressed interest in the more ambiguous (and perhaps less easily measurable) policy aims, i.e. establishing men's health needs, community engagement and support. A recent Canadian study found that despite public health professionals' awareness of the social determinants of health and beliefs about its importance, day-to-day their focus was determined by the need to demonstrate specific outcomes using measurable indicators, which in turn favoured an orientation towards behavioural interventions [84].

The primary interest in ‘intervention-as-outcome’ observed in this study provides some insight, and a possible partial explanation as to why such policy interventions have such a poor record in evidencing impacts on health outcomes.

### 4.5. Strength and weaknesses of the study

Our interviewees were predominantly health professionals and this might have explained the focus on ‘behavioural deficit’ explanations about the men's health problem as professionals often hold predominantly bio-medical, individualistic notions of health and illness [84],[85]. Social care professionals may well have identified other explanations about interventions [86].

Ideally, interviews with policy makers may have proved more informative. Despite gaining agreements from the relevant policy makers to be interviewed, those interviews did not take place as anticipated. It has been found elsewhere that policy makers are reluctant to take part in interviews about the policies they are responsible for [9]. Therefore, documentary records were the best representation of those available.

And, while the data for this study was collected in 2006, policy maker and professional interest in the use of rational planning frameworks by policy implementers for policy implementation has not abated. For example, the Royal Society for Public Health in the UK offers national training and resources in health planning for those involved in health improvement [52] whilst many health agencies in Scotland are specifically promoting an iteration of Logic Modelling, Outcome-Focused planning as the preferred means of planning and evaluating health improvement work [87],[88]. It seems unlikely that professional values, attitudes and practices associated with the implementation of policy intervention will have changed substantially over this period of time, and therefore these findings remain relevant to today's researchers, policy makers and implementers.

Furthermore, the voices and perspectives of men themselves were missing from this analysis of this policy interpretation/implementation process. Men were interviewed (as per the original evaluation commission) in respect of their views and experiences of the services and programmes that were offered as part of the WMS initiative only after they had been rolled out. Having those pre WMS design views would have provided valuable insights into the likely relevance and appeal the intended professional's plans would have had from the perspective of those intended to benefit from them.

The policy formulation process is (and remains) an intensely political, values-laden activity, characterised by dialogue involving a range of actors (such as policymakers, politicians, research scientists, special interest groups) who often hold competing perspectives, and have different levels of power with which to promote their individual viewpoints [89]. Galt (1994) argued that even if policy makers could use objective and logical processes to create policy (a notion strengthened in recent decades by the movement towards evidenced based policy-making), they are constrained by the complex, contested and politically ‘wicked’ nature of the problems they commonly face [11]. Such ‘wicked’ problems are consequently open to interpretation and debate, and unlike complex engineering or mathematical problems that rational linear thinking has been so successful in dealing with, have no one absolutely right or wrong solution [90]. There has been a tendency over many decades to think about public health problems, and the programmes intended to address them, as objective, fixed and bounded entities, when (as a growing number of health researchers argue) they should be regarded a complex and dynamic set of social processes [91]–[92]. Furthermore, those concerned with health equity research argue that the root causes of such inequalities are associated with politics and political ideology associated with the social determinants of health [78],[81],[93],[94]. Yet public health professionals are generally working within a paradigm that views health as individualised and depoliticised, where the role and influence of political ideology and values in health policy are implicit, but not acknowledged [95].

## 5. Conclusion

Public health professionals find themselves increasingly responsible for responding to centrally-determined, government health improvement policy initiatives, and for translating those into ‘real world’ interventions. This analysis indicated that if a purely rational, mechanistic planning heuristic is used to support the process of policy implementation, (as public health professionals are encouraged to do) they will continue to experience manifold challenges in effectively delivering any such policy effectively. We need to develop new ways of thinking about public health issues that have become ‘policy problems’ deemed in need of intervention and resolution, by governments, policy makers, public health professionals and researchers. A more honest dialogue is also required amongst these stakeholder groups which acknowledge the complex, contested and political nature of health problems and the policy implementation process. This would include accepting: (1) that a range of different perspectives and interpretations of public health policy problems and associated notions of their solutions is likely to reside amongst those individuals and organisations tasked with transforming policy into practice; (2) the inevitability of imperfect and contested evidence; and (3) the future uncertainties associated with trying to address complex public health issues.
